# The number of osteoblasts and osteoclasts in hypofunctional teeth during orthodontic tooth movement in rats

**DOI:** 10.12688/f1000research.53728.2

**Published:** 2022-04-29

**Authors:** Adibah Maulani, Cendrawasih Andusyana Farmasyanti, Darmawan Sutantyo

**Affiliations:** 1 Orthodontic Postgraduate Education Program, Faculty of Dentistry, Gadjah Mada University, Sleman, Yogyakarta, 55281, Indonesia; 2 Department of Orthodontics, Faculty of Dentistry, Gadjah Mada University, Sleman, Yogyakarta, 55281, Indonesia

**Keywords:** Tooth movement, osteoblast, osteoclast, hypofunctional

## Abstract

**Background:** When moved orthodontically, hypofunctional teeth will have a decreased tooth movement rate compared to normal teeth. This study aimed to determine the number of osteoblasts in the tension side and the number of osteoclasts in the pressure side of the hypofunctional teeth during orthodontic tooth movement. **Method:**18 male Wistar rats were given a palatal coil spring application on the maxillary incisors. Rats were divided into two groups, the orthodontic group with normal occlusion (NO) and hypofunctional occlusion (HO). The number of osteoblasts on the tension side and osteoclasts on the pressure side on days zero (D 0), five (D 5), and 10 (D 10) were tested with two-way ANOVA. Observations were made by hematoxylin eosin staining.

**Result:** The results showed that the number of osteoblasts on the tension side of the HO group was the same at the NO group (p> 0.05). The number of osteoblasts on the tension side in the NO and HO groups at D 5 was the same at D 10 (p = 0.99), but significantly higher (p = 0.002), than D 0. The number of osteoclasts on the pressure side in the HO group was significantly lower than the NO group (p <0.05). The number of osteoclasts in the NO D 5 group was significantly higher than the other groups (p <0.05).

**Conclusions:** The number of osteoblasts on the tension side was not affected by the hypofunctional state but decreased the number of osteoclasts on the pressure side during orthodontic tooth movement.

## Introduction

Tooth movement in orthodontic treatment is a biological response to mechanical forces characterized by remodeling processes in dental and paradental tissue, including pulp tissue, periodontal ligaments, alveolar bone, and gingiva ^1^. Osteoblasts, osteoclasts, and osteocytes play an essential role in bone remodeling in orthodontic tooth movement ^2^.

Clinicians often encounter cases which need to move the teeth that functionally never have occlusal pressure or hypofunctional teeth, such as open bite, ectopic canine, linguioversion and bucoversion teeth ^3^. Open bite malocclusion occurs when maxillary and mandibular teeth are not in contact ^4^.

Hypofunctional teeth cause atrophic changes in the periodontal ligament, a decrease in the number of periodontal fibers and blood vessels, and the periodontal space's narrowing. Periodontal space's narrowing occurs due to the apposition of the alveolar bone by an increase in Transforming Growth Factor β (TGFβ), causing tooth elongation ^5^. Changes in the paradental structure of hypofunctional teeth cause different reactions when orthodontically moved than normal teeth, especially in periodontal ligament tissue. Hypofunctional teeth when orthodontically moved have less heparan sulfate proteoglycan exposure, which plays a role in the osteoclastic activity, compared to normal teeth ^5^. Expression of Vascular Endothelial Growth Factor (VEGF) in hypofunctional teeth also decreases during orthodontic movement leading to vascular constriction and endothelial cell apoptosis. The expression of VEGF has an important role in the resorption and apposition processes of alveolar bone because it affects the proliferation and differentiation of osteoblasts and osteoclasts in vitro ^6^. Research on the number of osteoblasts on the tension side and osteoclasts on the pressure side in hypofunctional teeth during orthodontic movement has never been done before. This study aimed to determine the number of osteoblasts on the tension side and osteoclasts on the pressure side on hypofunctional teeth, respectively, during orthodontic tooth movement.

Wistar rats are considered a good research model for this study into orthodontic tooth movement because rats are cheap, making them easy to use as a large quantity sample, and the histological profiles of rats are easy to compare, especially in incisor teeth. Incisor teeth of Wistar rats have a gingival structure that almost resembles humans’, and is easy to install the orthodontic appliance into ^7^.

## Methods

### Ethical considerations

All experimental procedures were performed according to the Institutional Animal Care and Usage Committee (ARRIVE guidelines). The Ethical Clearance was approved by Ethical Committee of the Faculty of Dentistry of Universitas Gadjah Mada Yogyakarta, Indonesia, with Ethical Clearance number 00288/KKEP/FKG-UGM/EC/2019. All procedures involving rats were carried out with consideration to eliminate any suffering in the rats by using anesthetic drugs and euthanasia procedures during rats’ tissue collection.

### Animals

This study used 18 five-month-old, male, healthy rats weighing ± 400 grams, which had never been used in any procedures before. Rats adapted beforehand for seven days on a standard diet, including pellets. Rats were placed in cages at room temperature, which was 26C. Inclusion criteria related to body weight, sex, age, and health condition of the rats. Exclusion criteria included any technical issues that could disrupt orthodontic tooth movement, such as trapped bonding inside palatal coil.

Experimental animals were divided into two groups: the normal occlusion (NO) and the hypofunctional occlusion group (HO), both were moved orthodontically. This study was done without a control group in order to examine orthodontic tooth movement with and without occlusion over a period of time. In the HO group, the mandibular left incisors were cut to the gingival margin level every two days to obtain consistent spacing throughout the study. The sample size was determined using the Federer formula. Each group consisted of three rats with three groups of observation days: day zero (D0), day five (D5), and day 10 (D10). Rats were allocated to their groups using a simple randomization method: each rat was labelled, and a blindfolded researcher drew corresponding labels from a hat for each group. Researchers were aware of which group was which during the experiment.

### Procedures

**Figure 1.**
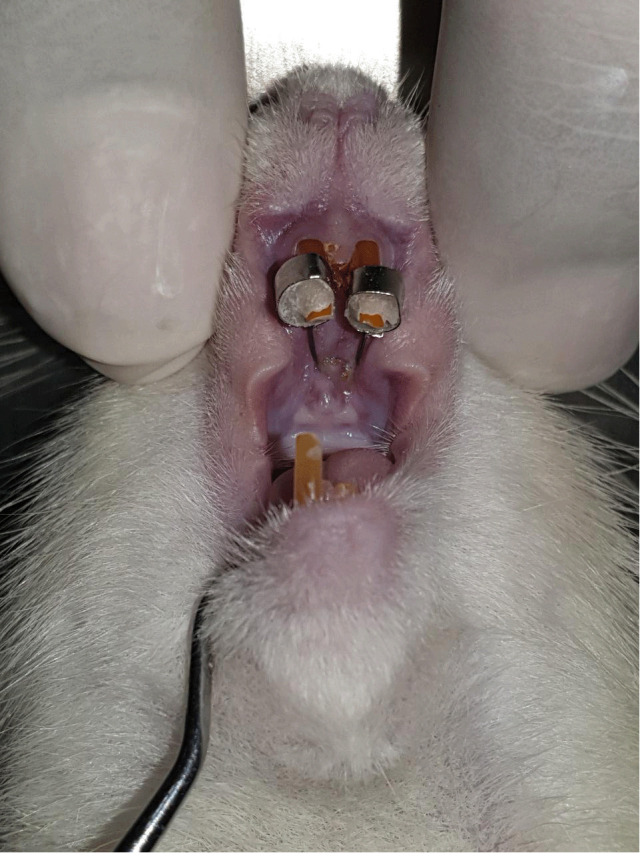
Installation of a palatal coil spring on rat incisors accompanied by reduction of lower incisors.

Animals were anesthetized using 10% ketamine 35 mg/kg and 2% xylazine 5 mg/kg intramuscularly during spring installation and reduction of left lower incisor. The upper incisors were separated using a customized palatal coil spring of 0.012 mm stainless steel wire (Ortho Prime Inc. USA: A 85021201; orthoshape SS 0,012”) connected to two metal bands (Dentaurum) with the arm length is 5 mm and the coil diameter is 2 mm. The customized coil spring was deflected for 3.4 mm to deliver an orthodontic force of 17.5 cN per upper incisor before being installed ^8^. The palatal coil spring was cemented using GIC Fuji IX, as shown in Figure 1. Then the left lower incisor was cut.

**Figure 2.**
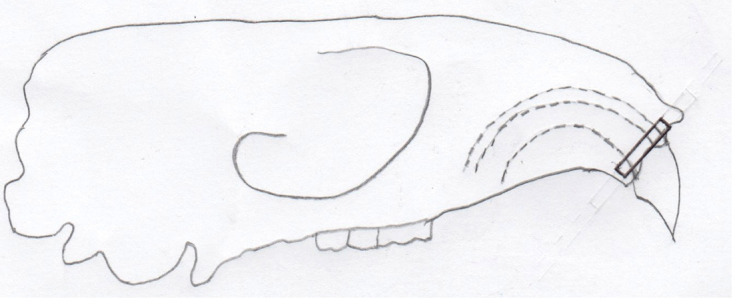
Alveolar crest region of the upper incisor where cross-section was taken shown in the black rectangle.

All experimental animals in day zero, day five, and day 10 groups were euthanized using an overdose solution of ketamine and xylazine (lethal dose: ketamine (KEPRO.BV production), 300 mg/kg BW and brand xylazine (Xyla) 30 mg/kg BW) intraperitoneally. Cross sections were taken on alveolar crest region of the upper incisor, shown in Figure 2. The number of osteoblasts were counted on the tension side and osteoclasts were counted on the pressure side using hematoxylin eosin staining and observed using an optical microscope (Olympus CX-22) with 400 times magnification in three fields of view every slide. Osteoblast cells appear cuboidal or columnar, purple, and single-nucleated ^9^. Osteoclast cells appear multinucleated with random boundaries, and purple in the resorption lacunae ^10^.

### Statisical analysis

The program used to perform statistical analysis was SPSS version 17.0 for Windows. Cohen's Kappa test value from two observers showed more than 0.50, which means there was good agreement between the two observers. The two observer were two-trained person who performed the measurement of osteoblast and osteoclast cell histologically. They were blinded to the applied sample. All data were normally distributed and homogeneous. The research data were then analyzed using the two-way ANOVA test followed by the Post Hoc test, Multiple Comparison (LSD). The confidence level used in this study is 95%.

## Results

The results in Table 1 show that the number of osteoblasts on the tension side of HO group is higher than NO group, but the difference is not significant (p = 0.187). The number of osteoblasts in NO group increased significantly on day five and continued to increase until day 10, as seen in Table 2, in contrast to HO group, which increased until day five but slightly decreased on day 10.

**Table 1.** Mean of osteoblasts on the tension side (cells/field).

Group	Mean ± SD		
	D _0_	D _5_	D _10_
NO	60.66 ± 9.50	112.33 ± 14.84	116 ± 18.24
HO	80.33 ± 9.50	124 ± 19.15	123 ± 38.93

Abbreviations: SD, standard deviations; NO, normal occlusion group; HO, hypofunctional occlusions group; D _0_, day zero; D _5_, day five; D _10_, day 10.

**Table 2.** Post Hoc LSD of osteoblasts on the tension side in each observation day.

Group	D _0_	D _5_	D _10_
D _0_	-	0.002*	0.002*
D _5_	-	-	0.990
D _10_	-	-	-

Abbreviations: D _0_, day zero; D _5_, day five; D _10_, day 10.

The highest number of osteoblasts on the tension side was seen in HO group on day five. The lowest number of osteoblasts on the tension side was seen in NOgroup on day zero.

**Figure 3.**
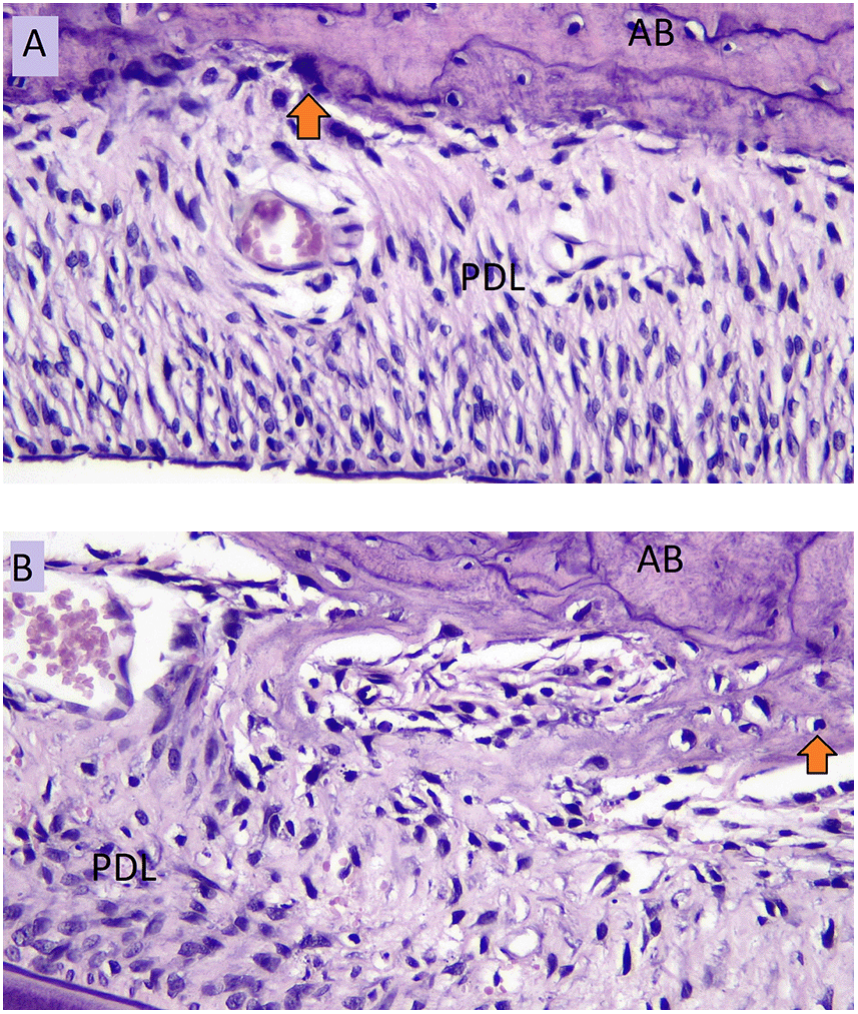
Osteoblast cells (arrow) of NO group ( **A**) and osteoblast cells of HO group ( **B**) in the tension side during orthodontic tooth movement. AB: Alveolar Bone, PDL: Periodontal Ligament.

**Figure 4.**
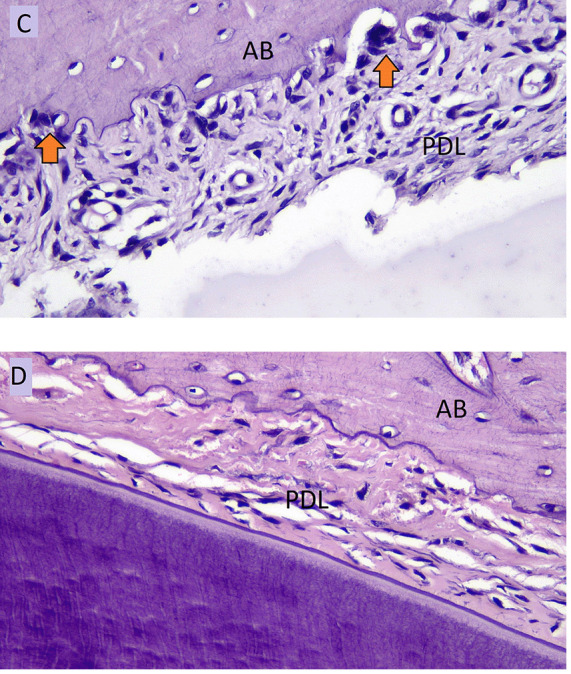
Osteoclast cells (arrows) of NO group ( **C**) and osteoclast cells of HOgroup ( **D**) in pressure side during orthodontic tooth movement. AB: Alveolar Bone, PDL: Periodontal Ligament.

The number of osteoblasts in NO group (A) and the number of osteoblasts in HO group (B) in the tension side during tooth movement is shown in Figure 3. Figure 4 showed the number of osteoclasts in NO (C) and HO groups (D) in the pressure side during orthodontic treatment.

The results in Table 3 showed that the number of osteoclasts in HO group was significantly lower than NO group on each day of observation (p = 0.014). The number of osteoclasts on the pressure side during orthodontic tooth movement in NO group began to significantly increase until day five, as seen in Table 4, then decreased on day 10. This pattern was the same as in HO group, which increased until day five, then decreased on day 10. The highest number of osteoclasts on the pressure side was seen in NO group on day five.

**Table 3.** Mean of osteoclasts on the pressure side (cells/field).

Group	Mean ± SD		
	D _0_	D _5_	D _10_
NO	1.33 ± 1.52	4 ± 2	0.67 ± 0.577
HO	0	1.33 ± 1.15	0

Abbreviations: SD, standard deviations; NO, normal occlusion group; HO, hypofunctional occlusions group; D _0_, day zero; D _5_, day five; D _10_, day 10.

**Table 4.** Post Hoc LSD of osteoclasts on the pressure side in each observation day.

Group	D _0_	D _5_	D _10_
D _0_	-	0.011*	0.626
D _5_	-	-	0.004*
D _10_	-	-	-

Abbreviations: D _0_, day zero; D _5_, day five; D _10_, day 10.

The results in Table 5 showed that rate of orthodontic tooth movement in NO group was increase from day zero to day 10, but the increase was not significant. The rate of orthodontic tooh movement in HO group was significantly increase from day zero to day five, but than decrease on day 10. The rate of orthodontic tooth movement on day 10 was higher in NO than HO group, but this difference was not significant.

**Table 5.** Mean of Orthodontic Tooth Movement Rate (mm).

Group	Mean ± SD		
	D _0_	D _5_	D _10_
NO	6.10±0,06	6.20±0,20	6.44±0,09
HO	5.78±0,36	6.66±0,23	6.06±0,20

Abbreviations: SD, standard deviations;NO, normal occlusion group; HO, hypofunctional occlusions group; D _0_, day zero; D _5_, day five; D _10_, day 10

## Discussion

The study showed that the number of osteoblasts in NO group had increased significantly on day five and then showed no significant difference until day 10, as seen in Table 2. This result was in line with Herniyati’s ^11^ research, which stated that the formation of preosteoblasts from mesenchymal cells had occurred 10 hours after applying force, followed by the differentiation of osteoblasts 40–48 hours later. The maximum number of osteoblast was reached on the 6th day of orthodontic tooth movement ^9^. This osteoblast differentiation and proliferation lasted up to 10 days ^13^.

The increasing number of osteoblasts on the tension side during 10 days of observation occurs because in the early phase of orthodontic tooth movement there will be an acute inflammatory response characterized by periodontal tissue vasodilation and prostaglandin secretion and growth factors such as TGFβ ^1^. TGFβ is also produced by fibroblasts on the tension side. TGFβ is an important factor in osteoblastogenesis and bone formation by recruiting osteoblast progenitors and stimulating the differentiation of bone matrix. An increase in TGFβ will increase osteoblast proliferation on the tension side ^14^. This acute inflammatory response will lead to an increasing number of osteoblasts in the early phase. One to two days later, the acute phase of inflammation is replaced by a chronic inflammatory process that is more proliferative, involving fibroblasts, endothelial cells, and osteoblasts ^1^.

The number of osteoblasts on the tension side during orthodontic movement of teeth with normal occlusion is influenced by several growth factors that are sensitive to mechanical stimuli, such as the expression of TGFβ, VEGF, Fibroblast Growth Factor (FGF), and Insulin-like Growth Factor (IGF). The increase in growth factor on the tension side will cause an increase in the number of osteoblasts. Hypofunctional teeth, without orthodontic force, will experience an increase in TGFβ expression, which simultaneously decreases VEGF, IGF, and FGF expression in the periodontal tissue ^5, 6, 15, 16^. Transforming Growth Factor β has a role in stimulating osteoblast differentiation and osteoclast apoptosis ^5^. Decreased FGF will lead to osteoblast differentiation in hypofunctional teeth because FGF works to inhibit osteoblast differentiation ^16^. The decrease in IGF causes a decrease in osteoblast proliferation because IGF is dominant in providing osteogenic effects ^17^.

Teeth that are hypofunctional when moved orthodontically will tend to experience decreased VEGF expression on both the tension and pressure sides ^6^. Decreased VEGF expression will cause apoptosis of endothelial cells, causing vascular constriction and decreased permeability. This will reduce the migration of osteoblasts on the tension side ^6^. Increased TGFβ and decreased FGF in hypofunctional teeth will increase osteoblasts.

The results showed the number of osteoblasts on the tension side of the hypofunctional teeth was the same as normal teeth during orthodontic movement (p > 0.05). This was possible because before orthodontic movement there was an increase in osteoblasts due to the interaction of increasing TGFβ and decreasing FGF and IGF, but simultaneously when hypofunctional teeth were given orthodontic force, there was a decrease in VEGF which tended to decrease osteoblast differentiation and migration, so that the number of osteoblasts became the same as the normal group. This needs further research.

The results showed the number of osteoclasts on the pressure side of NO group began to increase on the first day after the installation of a palatal coil spring and continued increasing until the fifth day, then decreased. on day 10. This result was almost the same as in the hypofunctional group, which increased up to day five, then decreased on day 10. This result is in line with the study by Miyoshi ^18^ which states that orthodontic movements immediately after force application are almost absent in osteoclasts. After the third day of mechanical strength application, several osteoclasts appeared. The maximum number of osteoclasts was reached on day six of orthodontic tooth movement ^12^. The increase in osteoclasts on day three was in line with the increase in VEGF expression, which also increased sharply ^19^.

An increasing number of osteoclasts occur because, in the early phase, the mechanical stress in the compression area will stimulate mechanoreceptors on osteocytes and cause changes in flow and blood vessels, causing tissue hypoxia that activates VEGF ^20^. VEGF plays an essential role in the angiogenesis process in the area of hyalinization ^19^. VEGF also plays a role in vascular permeability and activates endothelial cells. Active endothelial cells in the area of compression will cause chemoattraction of acute inflammatory cells such as leukocytes, monocytes, and macrophages. Leukocytes will stimulate prostaglandins and macrophage-colony stimulating factor (M-CSF). Increased prostaglandins in the area of compression will stimulate osteoblast differentiation and receptor activator of nuclear factor-kappa B ligand (RANKL) expression, whereas M-CSF can induce osteoclast differentiation by attaching to the c-Fms receptor on monocytic lineage cells. RANKL and M-CSF play an essential role in the process of osteoclast differentiation and bone resorption ^20^.

The number of osteoclasts on the pressure side in HO group was smaller than NO occlusion group on each observation day. This result was probably because VEGF expression in hypofunctional teeth decreases during orthodontic movement leading to vascular constriction and endothelial cell apoptosis ^6^. Endothelial cell apoptosis will cause decreased osteoclast differentiation and bone resorption ^20^. The decrease in VEGF will also cause a decrease in vascular permeability so that it will significantly imply a decrease in the number of osteoclasts ^21^.

Orthodontic tooth movement involves osteoblastic activity on the tension side and osteoclastic activity on the pressure side ^1^. The decrease in the number of osteoclasts on the pressure side in the orthodontic tooth movement of this HO group suggests a possible decrease in the rate of orthodontic tooth movement The research of Usumi-Fujita ^6^ states that there is a decrease in the rate of orthodontic movement in hypofunctional teeth. However, the rate of orthodontic tooth movement from this study showed that on day 10, there was no significant difference between HO and NO group. The rate of orthodontic tooth movement in hypofunctional teeth need further research.

## Conclusion

In conclusion, the number of osteoblasts on the tension side was not affected by the hypofunctional condition but decreased the number of osteoclasts on the pressure side during orthodontic tooth movement. The number of osteoclasts in HO group is lower compared to NO group during orthodontic tooth movement. It is possible that this is because of the decrease in VEGF and heparan sulfate proteoglycan.

## Data availability

### Underlying data

Figshare: The Number of Osteoclast and Osteoblast in Hypofunctonal Teeth during orthodontic tooth movement. https://doi.org/10.6084/m9.figshare.14515740.v10

This project contains the following underlying data:

- osteoclasts.xlsx
- osteoblasts.xlsx
- table of statistic analysis.docx
- Figure 1. jpg
- Figure 2. jpg
- Figure 3 A.jpg
- Figure 3 B.jpg
- Figure 4 C.jpg
- Figure 4 D.jpg

## Reporting guidelines

Figshare: ARRIVE Checklist, Maulani *et al*. https://doi.org/10.6084/m9.figshare.14515740.v8.

Data are available under the terms of the Creative Commons Zero “No rights reserved” data waiver (CC0 1.0 Public domain dedication).
