# Supplementary material for: The number of osteoblasts and osteoclasts in hypofunctional teeth during orthodontic tooth movement in rats
Source: F1000Res. 2022 Jun 13;10:541. Originally published 2021 Jul 6. [Version 3] doi: 10.12688/f1000research.53728.3 (PMC11587237; doi:10.12688/f1000research.53728.3)
Supplement: Peer review history

# Reviewer report: Reviewer response for version 3

**DOI:** 10.5256/f1000research.134578.r322700

**Published:** 2024-11-21

**Authors:** Mehmet Ali Darendeliler, Narayan Gandedkar

**Affiliations:** 1 Discipline of Orthodontics and Paediatric Dentistry, School of Dentistry, Faculty of Medicine and Health, The University of Sydney, Sydney, NSW, Australia; 2 School of Dentistry, Orthodontics, The University of Sydney Faculty of Medicine and Health (Ringgold ID: 522555), Sydney, New South Wales, Australia

This is a scientifically sound and good study and it has been reviewed twice, already. I have some points that will need further explanation. 

 Material and methodology

- Why was the study limited for 10 days? The rate of orthodontic tooth movement (OTM) would be affected if less days are selected instead of more. Although, osteoblast differentiation and proliferation might last up to 10 days (due to acute inflammatory response). It would be interesting in knowing whether the results of the current study remained the same beyond 10 days. This phenomenon should be explained in the context of days and OTM, and the hypofunctional condition.
- 17.5 cN used per incisor should be explained further regarding undermining resorption. The reference used in the study is for inflammatory response study. Generally, a very low force is used for OTM study.

Discuss the current limitations of the study.

- Other biomarkers for OTM
- compression–tension theory limitations in the light of other theories and current understanding of OTM
- Immunostaining Vs H & E staining (current study) method.

Authors need to add and discuss the following articles:

 Reference: 1, 2

Is the work clearly and accurately presented and does it cite the current literature?

Yes

If applicable, is the statistical analysis and its interpretation appropriate?

Yes

Are all the source data underlying the results available to ensure full reproducibility?

Yes

Is the study design appropriate and is the work technically sound?

Yes

Are the conclusions drawn adequately supported by the results?

Yes

Are sufficient details of methods and analysis provided to allow replication by others?

Yes

Reviewer Expertise:

Orthodontics, tooth movement, root resorption, obstructive sleep apnea, accelerated tooth movement, jaw orthopedics

We confirm that we have read this submission and believe that we have an appropriate level of expertise to confirm that it is of an acceptable scientific standard, however we have significant reservations, as outlined above.

## References

[1] : Effects of long-term occlusal hypofunction and its recovery on the morphogenesis of molar roots and the periodontium in rats. *Angle Orthod* .2013;83(4) : 10.2319/081812-661.1 597-604 10.2319/081812-661.1 23148606 PMC8754027

[2] : Open bite as a risk factor for orthodontic root resorption. *Eur J Orthod* .2013;35(6) : 10.1093/ejo/cjs100 790-5 10.1093/ejo/cjs100 23307804

# Reviewer report: Reviewer response for version 3

**DOI:** 10.5256/f1000research.134578.r339215

**Published:** 2024-11-19

**Authors:** Janvier Habumugisha

**Affiliations:** 1 Okayama University Graduate School of Medicine, Okayama, Japan

**Abstract**

**Thank you for the opportunity to review this paper. The author aimed to determine the number of osteoblasts on the tension side and osteoclasts on the pressure side of hypofunctional teeth during orthodontic tooth movement. The manuscript shows potential and is well-structured; however, the following points require further clarification:**

 Please ensure that all abbreviations are written out in full upon first usage. Specifically, in the abstract, define ' **VEGF**' before using the abbreviation. This will enhance clarity and accessibility for readers who may be unfamiliar with the terminology

 The sentence in abstract, the sentence, 'The results showed that the number of osteoblasts on the tension side of the HO group was the same at the NO group (p > 0.05),' please note that 'at' should be replaced with 'as in' to ensure grammatical accuracy. The corrected sentence would read: ‘ **The results showed that the number of osteoblasts on the tension side of the HO group was the same as in the NO group (p > 0.05)**.

 For clarity and better flow in the conclusion, I recommend revising the sentence as follows:' **The number of osteoblasts on the tension side was not affected by the hypofunctional state, but the number of osteoclasts on the pressure side decreased during orthodontic tooth movement**.

**Introduction**

 For clarity and readability, I suggest revising the second paragraph of the introduction as follows:

 ' **Clinicians often encounter cases that require the movement of teeth that have never experienced occlusal pressure or are considered hypofunctional. These cases include conditions such as open bites, ectopic canines, and lingually or bucally displaced teeth**.'

 I suggest rephrasing the paragraph in the introduction regarding the hypothesis as follows:' **Based on research on hypofunctional teeth, the following hypotheses were formulated: (1) the number of osteoblasts on the tension side of hypofunctional teeth is higher than in normal teeth during orthodontic tooth movement; (2) there is an increase in the number of osteoblasts and osteoclasts in hypofunctional teeth at both 5 and 10 days of orthodontic tooth movement; and (3) the number of osteoclasts on the pressure side of the alveolar bone in hypofunctional teeth is lower than in normal teeth during orthodontic tooth movement. Research on the number of osteoblasts on the tension side and osteoclasts on the pressure side in hypofunctional teeth during orthodontic tooth movement has not been conducted previously. This study aimed to investigate the number of osteoblasts on the tension side and osteoclasts on the pressure side of hypofunctional teeth during orthodontic tooth movement**.

 I suggest revising the paragraph for clarity and flow as follows:' **Wistar rats are considered an ideal research model for studies on orthodontic tooth movement due to their cost-effectiveness, which allows for the use of large sample sizes. Additionally, the histological profiles of rats, particularly in their incisor teeth, are easy to compare. The gingival structure of Wistar rat incisors closely resembles that of humans, and orthodontic appliances can be easily installed in these teeth**

**Materials and Methods section**

 I suggest revising the paragraph for clarity and flow as follows:

 ' **This study used 18 healthy, male, five-month-old Wistar rats, each weighing approximately 400 grams, that had never been involved in any prior procedures. Male Wistar rats were chosen to eliminate the potential influence of hormonal variations during orthodontic tooth movement [8]. The rats were allowed to adapt to their environment for seven days on a standard diet, including pellets. They were housed in cages at room temperature (26°C)**.

 There are several grammatical issues in the manuscript that may affect the clarity of your work. Work on  steps that will help enhance the readability and overall quality of your work.

**Other points that need clarification:**

 The study uses H&E staining for counting osteoblasts and osteoclasts, which is useful for general cell identification. However, for future work, I recommend considering the use of more specific methods, such as qPCR or immunohistochemistry (IHC), to assess osteoblast markers like Runx2, ALP, Osteocalcin, and BMP-2, as well as osteoclast markers like Cathepsin K, TRAP, and RANKL. These techniques could provide more detailed insights into the functional status and activity of these cells during orthodontic tooth movement.

Is the work clearly and accurately presented and does it cite the current literature?

Yes

If applicable, is the statistical analysis and its interpretation appropriate?

Yes

Are all the source data underlying the results available to ensure full reproducibility?

Yes

Is the study design appropriate and is the work technically sound?

Yes

Are the conclusions drawn adequately supported by the results?

Yes

Are sufficient details of methods and analysis provided to allow replication by others?

Partly

Reviewer Expertise:

Orthodontics, Biochemiistry and molecular dentistry

I confirm that I have read this submission and believe that I have an appropriate level of expertise to confirm that it is of an acceptable scientific standard, however I have significant reservations, as outlined above.

# Reviewer report: Reviewer response for version 3

**DOI:** 10.5256/f1000research.134578.r140697

**Published:** 2022-06-16

**Authors:** Erliera Sufarnap

**Affiliations:** 1 Department of Orthodontics, Universitas Sumatera Utara Fakultas Kedokteran Gigi, Medan, Indonesia

Dear Authors

 I congratulate all the authors for the complete explanation of the manuscripts and additional citations. I decide no more revision for the manuscript. Thank you.

Is the work clearly and accurately presented and does it cite the current literature?

Yes

If applicable, is the statistical analysis and its interpretation appropriate?

I cannot comment. A qualified statistician is required.

Are all the source data underlying the results available to ensure full reproducibility?

Yes

Is the study design appropriate and is the work technically sound?

Yes

Are the conclusions drawn adequately supported by the results?

Yes

Are sufficient details of methods and analysis provided to allow replication by others?

Yes

Reviewer Expertise:

cellulars in the orthodontic tooth movement research

I confirm that I have read this submission and believe that I have an appropriate level of expertise to confirm that it is of an acceptable scientific standard.

# Reviewer report: Reviewer response for version 1

**DOI:** 10.5256/f1000research.57143.r119271

**Published:** 2022-01-28

**Authors:** Masaru Yamaguchi

**Affiliations:** 1 Ginza Orthodontic Clinic, Tokyo, Japan

This paper describes that the number of osteoblasts and osteoclasts in hypofunctional teeth during orthodontic tooth movement in rats.

 Generally, this manuscript is interesting. However, there are some concerns as presented and some of these are discussed below.

**Major Comments:**

1. Was the force in this study optimal? The optimal force for molar tooth movement in rat may be 10g. The 25cN may induce an undermining bone resorption. In incisor, 17.5 cN force may be strong.
2. How much was the amount of tooth movement? Please add the results of them.
3. I think that research on Hypofunction is generally more suitable for molars, but how about it?
4. The authors concluded that the number of osteoblasts on the tension side was not affected by the hypofunctional state but decreased the number of osteoclasts on the pressure side during orthodontic tooth movement. However, this statement seems inconsistent. Did the number of osteoblasts on the tension side in hypofunction group also decreased?
5. I think that immunostaining such as TGF-b, VEGF, FGF, and IGF will be a better paper.

Is the work clearly and accurately presented and does it cite the current literature?

Yes

If applicable, is the statistical analysis and its interpretation appropriate?

Yes

Are all the source data underlying the results available to ensure full reproducibility?

Yes

Is the study design appropriate and is the work technically sound?

Partly

Are the conclusions drawn adequately supported by the results?

Partly

Are sufficient details of methods and analysis provided to allow replication by others?

Partly

Reviewer Expertise:

Dental, Orthodontics, bone metabolism, inflammation

I confirm that I have read this submission and believe that I have an appropriate level of expertise to confirm that it is of an acceptable scientific standard, however I have significant reservations, as outlined above.

# Response

**Published:** 2022-04-08

**Authors:** Adibah Maulani

**Affiliations:** Gadjah Mada University, Indonesia

Dear Dr. Masaru Yamaguchi

 Thank you for your kind assistance in reviewing our manuscript and providing us with valuable advices. Please allow us to comment as follows :

**Was the force in this study optimal? The optimal force for molar tooth movement in rat may be 10g. The 25cN may induce an undermining bone resorption. In incisor, 17.5 cN force may be strong.**

 Thank you very much for your insight. The force in this study was moderate force and already used in previous study about the rate of orthodontic tooth movement in rat incisor.

****

**How much was the amount of tooth movement? Please add the results of them.**

 Thank you very much for your suggestion. We already revised in new version by adding the rate of orthodontic tooth movement of this study.

****

**I think that research on Hypofunction is generally more suitable for molars, but how about it?**

 Hypofunctional research is usually done on molar because the hypofunctional state is easier to obtain by removing the upper molar teeth of rats. However, in this study, we chose incisors in order to facilitate the installation of orthodontic appliances and the anatomical structure of the periodontal tissue of the rat incisor was almost similar to humans. The hypofunctional state in this study was obtained by cutting the lower incisors of rats every two days.

****

**The authors concluded that the number of osteoblasts on the tension side was not affected by the hypofunctional state but decreased the number of osteoclasts on the pressure side during orthodontic tooth movement. However, this statement seems inconsistent. Did the number of osteoblasts on the tension side in hypofunction group also decreased?**

 The number of osteoblast on the tension side in hypofunction group was actually increase, but not significant. Because it was not significant, we conclude that the number of osteoblast on tension side was not effected.

****

**I think that immunostaining such as TGF-b, VEGF, FGF, and IGF will be a better paper.**

 Thank you very much for your suggestion. We would continue the study to know deeper using immunostaining.

# Reviewer report: Reviewer response for version 1

**DOI:** 10.5256/f1000research.57143.r89164

**Published:** 2021-07-15

**Authors:** Erliera Sufarnap

**Affiliations:** 1 Department of Orthodontics, Universitas Sumatera Utara Fakultas Kedokteran Gigi, Medan, Indonesia

Please allow me to congratulate the authors to perform this research study which I found quite interesting and useful as a reference for further study in the orthodontics field. We have been reviewing  the manuscript.

 However, I may require some clarifications on the following issues. My reviews recommend reconsideration to have some minor revisions which I will addressing the inquiries below.

**General comment:**

 The paper is well organized and easy to follow. It also well-written and well-structured but unfortunately there were had some typos, inconsistent of using words or abbreviation, had an ambiguous comprehension in reporting the analysis results, and many statement didn’t follow with references. I suggest the revision of the English grammar structures by an expert and also to the statistician to interpret the result.

**A. Abstract:**

**- Background:**

- “When moved orthodontically, hypofunctional teeth will have a decreased tooth movement rate compared to normal teeth” - This background already described the results indirectly which can correlated to lower osteoclast production and increased osteoblast. Please find another reason as a research gap which doesn’t comprehend to the OTM, maybe from the periodontal point of view.

**- Results:**

1.  Please describe the exact numbers of the p-value of each osteoblast and osteoclast, and compare mean between groups as the result.
2. Osteoblast: The post hoc analysis which had been analysed to “compare” between each time points (internally) for both groups and please further describe with statistically words and maintain the chronological time points, i.e., significantly increased at D0-D5, D0-D10 and not significantly changed at D5-D10.

**B. Introduction:**

1. At the last chapter, the research gap and the objective of the study clearly mentioned but We couldn’t see any hypothesis described in the introduction.
2. Describe the reference of why the animal model being used

**C**. **M&M:**

**1. Animals:**

- Please explain scientifically with refference the rationale to choose the male Wistar rats. The hypofunctional teeth could be happened to all genders.
- “…without a control group”; In my opinion, the NO already intervened as a control group.
- The teeth were cut to the gingival margin level every 2 days. Explain about the teeth and pulp anatomical condition. Please also mention why the teeth were cut every 2 days.

**2.** **Procedures:**

- Figure 1: We couldn’t see properly the palatal coil spring.
- Microscope (Olympus); please provide the type of the Olympus microscope.
- Please provide the reference which were mentioned for the osteoblast and osteoclast characteristics

**3.** **Statistical analysis:**

-  Who were the two observers? Please mention in the manuscript.

**D** . **Results:**

1. The post hoc analysis were  compared the differences between each time point for both HO and NO groups. Please describe those results based on the data, i.e, at D0 to D5, D0 to D10 and D5 to D10.
2. Please consistent of using “the group’s name”; with or without abbreviation. As if it will choose without abbreviation please further consistent of using complete group’s name. For some journal, they would prefer mention with abbreviation though.

**E. Discussion:**

1. At the first sentence for osteoblast discussion it discussed about time interval analysis, the author described only NO group unfortunately from Table 2 only had 1 result, did it mean that the statistic aimed to compare between time for both groups together? It supposed that the HO and NO increased together significantly at D0-D5 and D0-D10. Please, it would be more satisfy as if the author would ask the statistician whether the post hoc analysis results addressed for each group or for both group.
2. The first paragraph described all about osteoblast, but at the second last sentence  it described about osteoclast. It has been incompatible discussion.
3. The second and third paragraphs describes slightly about Growth factors. Please describes first from the general (all GF) and then followed to each GF discussion.
4. Typos found in the TGF abbreviation supposed to be TGFb.
5. Some discussion for several paragraph didn’t have references.
6. We couldn’t find any study’s limitation. As if the study were flawless it would be accepted.

**F. Conclusion:**

1. Please conclude the study based on the results.
2. Please be consistent in using  the groups name.

Is the work clearly and accurately presented and does it cite the current literature?

Yes

If applicable, is the statistical analysis and its interpretation appropriate?

I cannot comment. A qualified statistician is required.

Are all the source data underlying the results available to ensure full reproducibility?

Yes

Is the study design appropriate and is the work technically sound?

Yes

Are the conclusions drawn adequately supported by the results?

Yes

Are sufficient details of methods and analysis provided to allow replication by others?

Yes

Reviewer Expertise:

cellulars in the orthodontic tooth movement research

I confirm that I have read this submission and believe that I have an appropriate level of expertise to confirm that it is of an acceptable scientific standard.

# Response

**Published:** 2022-04-08

**Authors:** Adibah Maulani

**Affiliations:** Gadjah Mada University, Indonesia

Dear Dr. Erliera Sufarnap

 Thank you for your kind assistance in reviewing our manuscript and providing us with valuable advices. Please allow us to comment as follows :

**Background:**

**“When moved orthodontically, hypofunctional teeth will have a decreased tooth movement rate compared to normal teeth” - This background already described the results indirectly which can correlated to lower osteoclast production and increased osteoblast. Please find another reason as a research gap which doesn’t comprehend to the OTM, maybe from the periodontal point of view.**

 Hypofunctional teeth would have less VEGF expression and decreased heparan sulfate proteoglycan production during orthodontic tooth movement. The study about periodontal structure of hypofunctional teeth that moved orthodontically is never been done before.

**Results:**

**Please describe the exact numbers of the p-value of each osteoblast and osteoclast, and compare mean between groups as the result.**

 p-value of osteoblast between NO and HO group is 0.187

 p-value of osteoclast between NO and HO group is 0.014

**Osteoblast: The post hoc analysis which had been analysed to “compare” between each time points (internally) for both groups and please further describe with statistically words and maintain the chronological time points, i.e., significantly increased at D0-D5, D0-D10 and not significantly changed at D5-D10.**

 Osteoblast significantly increased at D0 to D5 with p-value 0.002, and so is at D0 to D10. However, at D5 to D10, the number of osteoblast is not significantly different, with p-value 0.99.

**B. Introduction:**

**At the last chapter, the research gap and the objective of the study clearly mentioned but We couldn’t see any hypothesis described in the introduction.**

 Hypotheses of the study are:

- The number of osteoblasts on the tension side of the hypofunctional teeth is higher than normal teeth during orthodontic tooth movement.
- There is an increase in the number of osteoblasts and osteoclast of the hypofunctional teeth during 5 and 10 days of orthodontic tooth movement.
- The number of osteoclasts on the pressure side of the alveolar bone in hypofunctional teeth lower than normal teeth during orthodontic tooth movement.

**Describe the reference of why the animal model being used**

 This study used animal model because we would like to see histologically during orthodontic tooth movement in hypofunctional teeth. The histological profiles of rats are easy to compare, especially in incisor teeth. Incisor teeth of Wistar rats have a gingival structure that almost resembles humans (Ren *et al.*, 2004). Thank you for your kind assistances. We would complete the references in new version of the article.

**C. M&M:**

**1. Animals:**

**Please explain scientifically with refference the rationale to choose the male Wistar rats. The hypofunctional teeth could be happened to all genders.**

 The reason why we choose male Wistar rats is because we avoid hormonal influences in female Wistar rats during orthodontic tooth movement.

**“…without a control group”; In my opinion, the NO already intervened as a control group.**

 Thank you very much for your insight.

**The teeth were cut to the gingival margin level every 2 days. Explain about the teeth and pulp anatomical condition. Please also mention why the teeth were cut every 2 days.**

 Wistar rats is a rodent, so the incisor teeth will erupt continuously. To maintain the space between maxillary incisor teeth and mandible incisor teeth, the teeth were cut to the gingival margin every 2 days. The teeth and pulp was still in good condition even after being cut every 2 days. This method was done based on previous study by Silva and Merzel (2004).

**2. Procedures:**

**Figure 1: We couldn’t see properly the palatal coil spring.**

 Thank you very much for your suggestion, I have revised it in recent article.

****

**Microscope (Olympus); please provide the type of the Olympus microscope.**

 Olympus CX-22 (Olympus, Germany).

**Please provide the reference which were mentioned for the osteoblast and osteoclast characteristics**

 Osteoblast characteristic was based on previous study by Alhasyimi *et al.,* (2018) which mentioned that osteoblast is cuboid cell with single and deep-blue nucleus. Osteoclast cells appear multinucleated with random boundaries, and purple in the resorption lacunae (Florencia-silva *et al.*, 2015). Thank you for your kind assistances. We would complete the references in new version of the article.

**3. Statistical analysis:**

**Who were the two observers? Please mention in the manuscript.**

 The two observer were two-trained person who performed the measurement of osteoblast and osteoclast cell histologically. They were blinded to the applied sample. Thank you very much for your suggestion, I have revised it in recent article.

**D. Results:**

**The post hoc analysis were compared the differences between each time point for both HO and NO groups. Please describe those results based on the data, i.e, at D0 to D5, D0 to D10 and D5 to D10.**

 Osteoblast significantly increased at D0 to D5 with p-value 0.002, and so is at D0 to D10. However, at D5 to D10, the number of osteoblast was not significantly different, with p-value 0.99.

 Osteoclast significantly increased at D0 to D5 with p-value 0.011. However, osteoclast significantly decreased at D5 to D10 with p-value 0.004. The number of osteoclast between D0 to D10 was not significantly different, with p-value 0.626.

**Please consistent of using “the group’s name”; with or without abbreviation. As if it will choose without abbreviation please further consistent of using complete group’s name. For some journal, they would prefer mention with abbreviation though.**

 Thank you very much for your suggestion, I have revised it in recent article.

**E. Discussion:**

**At the first sentence for osteoblast discussion it discussed about time interval analysis, the author described only NO group unfortunately from Table 2 only had 1 result, did it mean that the statistic aimed to compare between time for both groups together? It supposed that the HO and NO increased together significantly at D0-D5 and D0-D10. Please, it would be more satisfy as if the author would ask the statistician whether the post hoc analysis results addressed for each group or for both group.**

 Thank you very much for your suggestion. The result between HO and NO group is not significantly different. Increasing number of osteoblast from D0 to D5 and then D10 on HO group has the same pattern with NO group, so the Post Hoc analysis is aimed to compare between time for both group together.

**The first paragraph described all about osteoblast, but at the second last sentence it described about osteoclast. It has been incompatible discussion.**

 Thank you for your kind assistance **, **I apologize for the typo. I have revised it in recent article.

**The second and third paragraphs describes slightly about Growth factors. Please describes first from the general (all GF) and then followed to each GF discussion.**

 Growth factors are proteins that attach to receptors on the cell surface, thereby activating a signal transduction, and subsequently affecting cell proliferation, differentiation, and apoptosis. Most growth factors have a specific effect on certain cell types on the process of proliferation and differentiation. For example, Fibroblast Growth Factor (FGF), Transforming Growth Factor (TGF)-β, and Insulin-like Growth Factor (IGFs), required for growth of bone, muscle, and other cells (Stone *et al.*, 2022).

  TGF-β (Transforming Growth Factor β) is growth factor that can affect bone metabolism. This mediator regulates bone remodeling by modulating osteoblasts and osteoclasts (Fox and Lovibond, 2005).

 Transforming Growth Factor has the role of stimulating differentiation osteoblasts and osteoclast apoptosis (Itohiya *et al*., 2016).

 FGF (Fibroblast Growth Factor) is a representative growth factor which has shown the potential effects on the repair and regeneration of tissues (Moya et al, 2010).

 Basic fibroblast growth factor (bFGF) also involved in stimulation of osteoblasts and osteoclasts (Seifi *et al.,* 2013).

 IGF, induced by growth hormone and parathyroid hormone, is reproduced by osteoblast, chondrocytes and other bone cells, and plays an essential part as a local modulator in cell differentiation and proliferation in either an autocrine or paracrine manner. Igf-I stimulates not only osteoblast proliferation but also osteoclast formation (Tokimasa *et al.*, 2003).

 VEGF (Vascular Endhotelial Growth Factor) is an essential mediator during the process of angiogenesis, bone remodelling by stimulating osteoblast differentiation, and osteoclastic recruitment (Yang *et al.*, 2012)..

**Typos found in the TGF abbreviation supposed to be TGFb.**

 Thank you very much for your suggestion, we have revised the typos in new version of the article.

**Some discussion for several paragraph didn’t have references.**

 Thank you for your kind assistances. We would complete the references in new version of the article.

**We couldn’t find any study’s limitation. As if the study were flawless it would be accepted.**

 Thank you for your kind suggestions, this study’s limitations including days of observations which was limited for 10 days. Further study should provide longer time, at least 14 days up to 21 days, to observe further effect of hypofunctional condition in the number of osteoblast and osteoclast cell during orthodontic tooth movement.

**F. Conclusion:**

**Please conclude the study based on the results.**

 The number of osteoblasts on the tension side was not affected by the hypofunctional condition but the number of osteoclasts on the pressure side was lower during orthodontic tooth movement under hypofunctional condition.

**Please be consistent in using the groups name**

 Thank you very much for your suggestion, we have revised the groups name recent article.

**References**

 Stone WL, Leavitt L, Varacallo M. Physiology, Growth Factor. StatPearls Publishing. 2022. https://www.ncbi.nlm.nih.gov/books/NBK442024/

 Fox SW, Lovibond AC. Current insights into the role of transforming growth factor-beta in bone resorption. *Mol Cell Endocrinol. *2005;243(1-2):19–26. 

  Itohiya K, Kazaki H, Ishikawa M, et al.: Occlusal Hypofunction Mediates Alveolar Bone Apposition via Relative Augmentation of TGF-β Signaling by Decreased Asporin Production in Rats. *Dental Oral Craniofacial Res.* 2016;3(1):1–8.

 Moya ML, Cheng MH, Huang JJ, Francis-Sedlak ME, Kao SW, Opara EC, Brey EM

 Biomaterials. 2010 Apr; 31(10):2816-26.

 Seifi M, Badiee MR, Abdolazimi Z, Amdjadi P. Effect of basic fibroblast growth factor on orthodontic tooth movement in rats. *Cell J*. 2013;15(3):230-237.

 Tokimasa, C., Kawata, T., Kaku, M., Kohno, S., Tsutsui, K., Tenjou, K., Ohtani, J., Motokawa, M., Tanne, K., 2003, Effects of Insulin-like Growth FactorI on the Expression of Osteoclasts and Osteoblasts in the Nasopremaxillary Suture under Different Masticatory Loading Conditions in Growing Mice, *Arch Oral Biol*, 48:31-8

 Yang YQ, Tan YY, Wong R, Wenden A, Zhang LK, Rabie AB. The role of vascular endothelial growth factor in ossification. Int J Oral Sci. 2012 Jun;4(2):64-8. doi: 10.1038/ijos.2012.33. PMID: 22722639; PMCID: PMC3412670.
